# Radiological appearance of hepatocellular carcinoma predicts the response to trans-arterial chemoembolization in patients undergoing liver transplantation

**DOI:** 10.1186/s12885-019-6265-1

**Published:** 2019-11-05

**Authors:** Wei Zhang, An-Hui Xu, Wei Wang, Yan-Hui Wu, Qian-Ling Sun, Chang Shu

**Affiliations:** 1 Hepatic Surgery Center, Tongji Hospital, Tongji Medical College, Huazhong University of Science and Technology, 1095 Jiefang Avenue, Wuhan, 430030 China; 2 Department of Radiology, Tongji Hospital, Tongji Medical College, Huazhong University of Science and Technology, Wuhan, China; 3 Surgery administrator office, Tongji Hospital, Tongji Medical College, Huazhong University of Science and Technology, Wuhan, China

**Keywords:** Hepatocellular carcinoma, Trans-arterial chemoembolization, Liver transplantation, Computed tomography, Necrosis

## Abstract

**Background:**

The ultimate goal of locoregional therapy (LRT) to the liver is to induce total tumor necrosis. Trans-arterial chemoembolization (TACE) is the mainstay bridging therapy for patients with hepatocellular carcinoma (HCC) waiting for liver transplantation (LT). However, tumor response rate is variable. The purpose of this study was to correlate HCC radiological appearance with level of tumor necrosis during explant analysis from patients undergoing LT who received pre-LT TACE.

**Methods:**

From January 2000 to December 2018, a total of 66 patients with HCC who had been treated prior to LT by means of TACE were analyzed. Diagnosis of HCC was made based on AASLD guidelines and confirmed via histopathology explant analysis. Radiologic tumor response after TACE was based on modified Response Evaluation Criteria in Solid Tumors (mRECIST). Degree of tumor necrosis was determined by histopathology analysis of liver explants. HCC radiological appearances on CT before TACE were assessed and correlated with histological findings after LT.

**Results:**

Eighty nine TACE procedures (1.35 ± 0.67; 1–4) were performed, of which 18 were repeated TACE (27.3%) procedures. In 56.1% of the patients, ≥90% (near-complete) tumor necrosis was achieved. Concordance between mRECIST criteria and pathology was observed in 63% of the patients, with an underestimation of tumor response in 18 (27%) patients and an overestimation in 6 (9.1%). Near-complete tumor necrosis upon pathological analysis was associated with tumor hyper-enhancement in the arterial phase (*P* = 0.002), “typical tumor enhancement” (*P* = 0.010) and smooth tumor margins (*p* = 0.011). The multivariate analysis showed that well circumscribed HCCs with smooth margins and arterial hyper-enhancement independently correlated with post-TACE near-complete histological tumor necrosis.

**Conclusions:**

The well circumscribed HCC lesions with arterial hyper-enhancement are more susceptible to TACE than lesions with arterial phase iso or hypo-enhancement and lesions with infiltrative appearance. Pre-TACE CT imaging may ease the selection of an optimal treatment strategy for bridging patients with HCC to liver transplantation.

## Background

Hepatocellular carcinoma (HCC) is the most common primary liver malignancy as well as a worldwide leading cause of cancer mortality [1]. With most HCCs arising with a background of cirrhosis, liver transplantation (LT) stands as the most important potentially curative therapy [2]. However, a major drawback of LT is the significant scarcity of donors. As a result, increases in waiting time have led to 20% of transplant candidates dropping out of the transplant waitlist [3]. Locoregional therapy (LRT) as a bridging strategy for patients on the waitlist aims to prevent tumor progression and shrink tumors to maintain transplant eligibility [4, 5]. Among other LRT, trans-arterial chemoembolization (TACE) has been the most commonly implemented modality of treating patients with HCC not amenable to resection [6]. Considering that incomplete necrosis can be a risk factor for post-LT HCC recurrence [7], the ultimate goal of TACE is to induce total tumor necrosis.

Assessment of tumor response to TACE via contrast enhanced (triphasic) cross-sectional imaging is critical in determining the success of treatment and in guiding future therapy. Modified response evaluation criteria in solid tumors (mRESIST) considers the size of viable contrast-enhancing areas within the tumor to evaluate response to LRT [8]. Unfortunately, accurate correlation of tumor size, number, percentage of necrosis and response to LRT between cross sectional imaging and histopathology is an ongoing challenge with 20–40% radiologic underestimation of tumor burden [9].

Although several studies have confirmed the correlation of the enhancement patterns and morphological image findings of HCC with tumor differentiation [10–12], there is limited data regarding the correlation of the imaging characteristics of HCC lesions before and after TACE with pathological analysis. It has been suggested that complete retention of iodized oil in the tumor tends to correlate to complete tumor necrosis and improved survival [13]. Patients that demonstrated no relevant contrast enhancement on the post-TACE CT had significantly more extensive necrosis [14, 15]. Nevertheless, it is still unknown if the pre-TACE radiological appearances of HCC correlate with degree of tumor necrosis.

The purpose of this study was to correlate the pre-TACE radiological appearance of HCC assessed via CT with the level of tumor necrosis upon histopathological examination of the explanted liver after liver transplantation.

## Methods

### Study populations

**Fig. 1:**
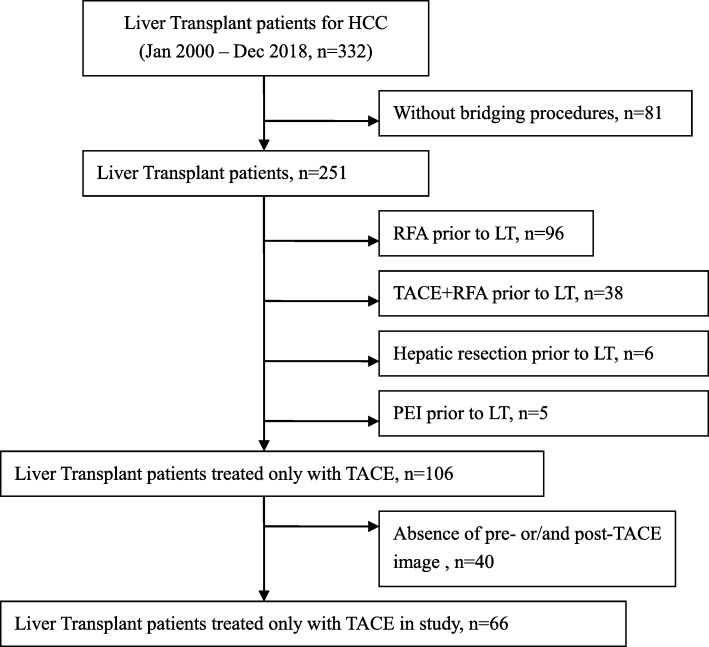
Flow chart of the study population

A search was conducted in a prospectively maintained LT database between January, 2000 and December, 2018. Among 332 patients undergoing LT for HCC, 81 patients without bridging procedures, 139 patients treated with other locoregional therapy [radiofrequency ablation (RFA), TACE+RFA and percutaneous ethanol injection (PEI)] and 6 patients receiving hepatic resection prior to LT were excluded. We also excluded 40 patients without pre- and/or post-TACE imaging. The final study population consisted of 66 patients treated only with TACE (Fig. 1). Diagnosis of HCC was made on histologic or radiologic criteria according to published guidelines [16] and confirmed on explant histopathology analysis. Percutaneous biopsy for diagnosis of HCC was not routinely performed.

In patient recruitment, the Milan Criteria was used as the primary indication of LT for HCC, in accordance with institution policy. After confirmation of diagnosis, patients supposed to be within the Milan Criteria were immediately listed for LT. LRT was performed in patients beyond Milan Criteria at initial imaging for downstaging, or in patients within Milan Criteria, but with an expected waiting time for bridging of more than 2 months. Those exceeding the Milan Criteria were listed after LRT if the treatment resulted in stable diseases or partial response according to the mRECIST criteria at 3 month intervals. Pre-transplant LRT include conventional TACE, RFA, TACE plus RFA, and percutaneous ethanol injection (PEI). TACE has been employed as a first line therapy in the patients with extensive disease burden (largest tumor size > 3 cm and/or tumor number > 3). Percutaneous radiofrequency ablation (RFA) is mainly used in lesions measuring≤3 cm. Patients were excluded from TACE if they had decompensated cirrhosis (Child-Pugh B, score > 8), severely reduced portal vein flow, extensive tumor with massive replacement of both lobes, renal insufficiency (creatinine ≥2 mg/dL or creatinine clearance ≤30 mL/min) and untreatable arterio-venous fistula [17]. In all cases, data from both the initial CT investigation and the last CT scan after TACE before LT, if available, were evaluated.

### CT technique and image evaluation

The pre- and post TACE multiphasic contrast enhanced studies of the enrolled subjects were performed on multidetector computed tomography (MDCT) scanners (ranging from 8 to 64 rows of detectors), with 0.6–1.5 -mm collimation, 3 -mm slice interval and 3–5 -mm slice thickness. Patients were scanned before and after the intravenous administration of iodine contrast media in the arterial phase, portal-venous phase and equilibrium phase. The studies were retrospectively reviewed on commercially available workstations (MV1000, Siemens Healthcare, and IMPAX, Agfa Healthcare) simultaneously by two expert abdominal radiologists, who were unaware of the explant histopathology findings.

The largest diameter of the enhancing portion of the lesions on pre-TACE CT scan was measured in the imaging phase in which the lesion was best seen, and only lesions greater than or equal to 1 -cm were included in the study. The HCC attenuation in each phase was classified as hyper-attenuation, iso-attenuation or hypo-attenuation, when compared with the surrounding liver parenchyma. A lesion with heterogeneous enhancement was regarded as hyper-attenuating when most of it was enhanced during the arterial phase compared with the pre-contrast phase. Hyper-attenuation on the arterial phase followed by washout in the portal or equilibrium phase was defined as “typical enhancement”. A hypo-attenuated area with no change in the degree of attenuation during the dynamic phase was defined as necrosis. The shape of the tumor margin was categorized into three subgroups: smooth; lobulated or infiltrative. Capsule appearance means a rim of hyper-enhancement in portal, delayed, or transitional phase, which is unequivocally thicker than fibrotic tissue around background nodules [18].

HCC lesions were classified in three major categories based on their pattern of enhancement and margins. Type A: Well-defined (or circumscribed) tumors with arterial phase hyper enhancement (relative to the background liver parenchyma) and portal-venous or equilibrium phase wash-out. Type B: Well-defined (or circumscribed) tumors with arterial phase iso- or hypo enhancement (relative to the background liver parenchyma) and portal-venous or equilibrium phase wash-out. Type C: Poorly defined (or infiltrative) tumors, irrespective of their enhancement pattern.

CT follow-up was performed 1 month after TACE, and at least every 3 months until LT. The post-TACE CT scan images performed prior to the liver transplant were subsequently reviewed in the same session and compared side-by-side with the pre-TACE CT scan, in order to assess the treatment response. The responses to TACE were categorized according to the mRECIST [19] as follows: complete response (CR), which included the disappearance of any intratumoral arterial enhancement; partial response (PR), which displayed a ≥ 30% decrease in the diameter of the viable portion (enhancement in the arterial phase) of a target lesion (taking the baseline diameter of the target lesion as a reference); progressive disease (PD), which was defined as a ≥ 20% increase in the diameter of the viable portion of a target lesion (taking as reference the baseline diameter of the target lesion); and stable disease (SD), which included any cases not categorized as PR or PD.

### TACE protocol

TACE was performed after patients provided written informed consent. 5-F catheters or 3-F coaxial microcatheters were inserted into the common femoral artery and angiographic survey of the celiac and superior mesenteric arteries was performed. Common hepatic angiography was performed to determine tumor blood supply, and then a microcatheter was selectively inserted into the artery feeding the tumor. We selected epirubicin (Pharmorubicin, Pfizer; Wuxi, Jiangsu, China) as the anticancer drug for suspending in Lipiodol. Epirubicin (20-40 mg) was dissolved in 2 mL saline and suspended in the adapted amount of Lipiodol (2–10 ml) (Guerbet, Roissy, France). The emulsion was injected into the supplying artery until stop of flow. Under fluoroscopic guidance, the vessels were subsequently embolized with Gelfoam (ALICON Pharm SCI &TEC CO., Hangzhou, China) until complete flow stagnation was achieved. The interventional radiologist chose the amount of the chemotherapeutic and embolic agents depending on the tumor size, number of tumors, tumor arterial blood supply, degree of liver impairment, and renal function. Repeat TACE treatment was typically scheduled in patients with adequate hepatic function reserve (Child-Pugh A or Child-Pugh B < 8) and residual viable tumor at 6 to 8 week intervals after the initial treatment. In contrast, in patients with no evidence of residual viable disease (i.e., with CR according to mRECIST), imaging follow-up was recommended every 2 to 3 months. If PR or SD was achieved, the patients were administered with the same chemotherapeutic agent. If progressive disease (PD) occurred, the chemotherapeutic agent was adjusted to cisplatin or lobaplatin.

### Histopathology

After transplantation, an experienced hepatopathologist performed the gross and histologic analyses of all explanted livers. The freshly explanted livers were sliced serially at 10-mm intervals. Routine hematoxylin and eosin staining were used to prepare the slides. The number, size, location and gross characteristics of all lesions were assessed. The grade of differentiation was based on the Edmondson and Steiner criteria (grade 1, well differentiated; grade 2, moderately differentiated; grade 3, poorly differentiated). The presence of lymphovascular invasion was recorded. If heterogeneous differentiation was found in the obtained tumor, differentiation grade was classified based on the lowest differentiated grade. The presence of partial necrosis was assessed by the pathologist as the percentage of necrotic tissue divided by total tumor tissue. A necrosis of 100% was assumed to indicate complete necrosis.

### Statistical analysis

Continuous variables were expressed as means and standard deviations, medians and ranges, or both. Categorical variables were reported as numbers and percentages. Pearson’s chi-square tests or Fisher’s exact tests were performed to evaluate categorical variables and the Student’s t-test for continuous data. Continuous variables were transformed into binary variables and the cutoffs were chosen according to previous studies. After univariate analysis, only variables that emerged as significant were used in the multivariate analysis using Cox’s proportional hazard model. Sensitivity, specificity, positive (PPV) and negative (NPV) predictive values, and accuracy of CT in the detection of 100% necrosis were calculated. A two-tailed *P*-value of < 0.05 was considered to be statistically significant. Statistical analyses were performed with SPSS 22.0 (SPSS, Inc., Chicago, IL) for Windows.

## Results

### Characteristics of patients and tumors

Patient characteristics are summarized in Table 1. The median age was 61 years (range, 25–72), and the ratio of males to females was 48:18 (72.7%:27.3%). Of the 66 patients, 50 (75.8%) patients were infected with hepatitis B virus (HBV). Child–Pugh class for liver function was class A in 49 patients (74.2%) and class B in 17 patients (25.8%). 68.2% of the patients had early–stage (stage A) cancer based on the Barcelona Clinic Liver Cancer staging system. At the time of treatment, 1.5% of disease was UNOS stage T1; 59.1%, stage T1; 24.2%, stage T3; 15.2% stage T4a. The median serum AFP levels were 13.7 ng/ml (range 2.3 to 4225.5). Forty-eight patients had a single course of TACE (72.7%), and the remaining 18 had two or more courses (27.3%). The mean number of TACE procedures was 1.35 ± 0.67 (range, 1–4). Mean tumor size was 3.43 ± 1.38 cm (1.3–8.0 cm) in the initial CT scan and 2.68 ± 1.15 cm (0.5–5.7 cm) in the last scan before transplant (*p* = 0.001).

**Table 1:** Patient Demographics

Variable	Value
Age (y), median (range)	61(25–72)
Gender, n(%)	
Male	54(81.8%)
Female	12(18.2%)
Etiology, n(%)	
HBV	50(75.8%)
HCV	6(9.1%)
HBV + HCV	5(7.6%)
Alcohol	3(4.5%)
Others	2(3%)
Child-Pugh class, n(%)	
A	49(74.2%)
B	17(25.8%)
BCLC class, n(%)	
A	45(68.2%)
B	21(31.8%)
MELD, median (range), n(%)	9.7 ± 3.0/9.0(6.0–19.0)
Within Milan criteria, n(%)	40(60.6%)
Total bilirubin (mg/dL), median (range)	1.2(0.3–8.8)
albumin(g/dL), median (range)	3.4(1.6–4.5)
INR (sec), median (range)	1.1(1.0–1.6)
ALT(U/L), median (range)	57.0(12.0–400.0)
Serum creatinine (mg/dL), median (range)	0.79(0.47–1.37)
AFP (ng/mL), median (range)	13.7(2.3–4225.5)
Time on transplant list (day), median (range)	97(31–509)
Time from first TACE to transplantant (day), median (range)	83(30–582)
TACE accomplished, Total no. (mean ± SD; range)	89(1.35 ± 0.67; 1–4)
Repeat TACE, n(%)	18(27.3%)

*Abbreviation*: *HBV* hepatitis B virus, *HCV* hepatitis C virus, *ETOH* ethanol alcohol, *NASH* nonalcoholic steatohepatitis, *BCLC* Barcelona Clinic Liver Cancer, *MELD* The Model for End-Stage Liver Disease, *INR* international normalized ratio, *ALT* alanine aminotransferase, *AFP* α-fetoprotein

Overall, a total number of 89 TACE procedures were accomplished. The median time from the first TACE procedure to LT was 83 days (range = 30–582 days), and the median time on the waiting list was 97 days (range = 31–509 days). All patients underwent lipiodol-TACE.

At histopathology examination (Table 2), 33 (50%) patients had single HCC, 11 (16.7%) patients had 2, 13 (19.7%) patients had 3, and 9 (13.6%) patients had more than 3 lesions. One hundred thirty-eight lesions were found in 66 liver explants. The mean number of lesions was 2.09 ± 1.49 (range 1–9). The mean size of the largest lesion was 2.93 ± 1.53 cm (0.6–7.0 cm). Tumors were graded as well, moderately, and poorly differentiated in 18.2, 75.8 and 6.0% of patients, respectively. Microscopic vascular invasion was detected in 14 (21.2%) patients.

**Table 2:** Tumor characteristics in histopathlogy

Variable	Value
Single lesion, n(%)	33(50%)
Multiple HCC, n(%)	33(50%)
Number of lesions, total no. (mean ± SD; range)	138(2.09 ± 1.49; 1–9)
Size of lesion (cm), median (range)	2.6(0.6–7)
Single nodule	2.6(0.8–6.5)
Multiple nodule	2.5(0.6–7.0)
Location, n(%)	
right	37(56.1%)
left	11(16.7%)
both	18(27.2%)
Differentiation, n(%)	
well	12(18.2%)
moderate	50(75.8%)
poor	4(6.0%)
Vascular invasion, n(%)	14(21.2%)
pTNM stage, n(%)	
T1	1(1.5%)
T2	39(59.1%)
T3	16(24.2%)
T4a	10(15.2%)

*Abbreviation*: *pTNM* pathologic tumor-node-metastasis stage

### Comparison of tumor necrosis according to mRECIST and histopathology

At explant histopathology analysis, the mean degree of tumor necrosis was 71.9 ± 32.3%. Tumor necrosis was rated as 0–30%, 31–60%, 61–90%, 91–99, and 100% in 10 (15.2%), 13 (19.6%), 17 (25.8%), 5 (7.6%), and 21 (31.8%) patients, respectively. The objective response rate (define as CR and PR) at CT was 92.4%, with 43.9% CR (29/66 patients), 48.5% PR (32/66 patients), 7.6% SD (5/66 patients). Comparison between mRECIST and histopathologic necrosis is presented in Table 3. A statistically significant relation was observed between mRECIST and pathologic necrosis (*r* = 0.550, *P* = 0.000).

**Table 3:** CT-pathology correction: mRECIST versus pathologic necrosis

CT (mRECIST)	NO.patients	Pathology necrosis(%)		
		< 30	30–99	100
SD	5/66	4	1	0
PR	32/66	5	22	5
CR	29/66	1	12	16

*Abbreviation*: *SD* stable disease, *PR* partial response, *CR* complete response, *mRECIST* modified RECIST

Assuming the correlation between 100% necrosis and CR, 30–99% necrosis and PR, < 30% necrosis and SD, the accuracy of mRECIST to assess tumor response was 63.6%, with 6 lesions (9.1%) of underestimation and 18 lesions (27.3%) of overestimation. CT sensitivity, specificity, PPV and NPV in identifying 100% tumor necrosis were 76.2, 71.1, 55.2, 86.5%, respectively.

### Predictors of near-complete necrosis

For further evaluating the variables related to pathologic necrosis, two subgroups were defined: near-complete necrosis ≥90% (*n* = 37) and necrosis < 90% (*n* = 29). The univariate analysis of the demographic and pathological variables related to near-complete necrosis was summarized in Table 4. The degree of necrosis correlated significantly with pre-TACE liver function (*p* = 0.045). In comparison to Child-Pugh class A patients, Child-Pugh class B patients had a lower percent of near-complete necrosis. The degree of necrosis did not correlate with initial tumor size (*p* = 0.641) and number (*p* = 0.219). No other patient or lesion characteristics were significantly associated with near-complete necrosis.

**Table 4:** Univariate analysis of the demographic and pathological variables related to near-compete necrosis

Variables	Tumor necrosis < 90%	Tumor necrosis ≥90%	Chi-square
Sex			
Male	24	24	*P* = 0.105
Female	5	13	
Age			
≤ 60 years	11	19	*P* = 0.277
> 60 years	18	18	
Etiology			
HCV positive	14	25	*P* = 0.114
HCV negative	15	12	
AFP			
≤ 200 ng/dL	23	33	*P* = 0.444
> 200 ng/dL	6	4	
Child-Pugh class			
A	18	31	*P* = 0.045
B	11	6	
Milan criteria			
Within	20	19	*P* = 0.149
Beyond	9	18	
Waiting time			
0–6 months	26	31	*P* = 0.743
> 6 months	3	6	
Time between first TACE and LT			
< 3 months	15	20	*P* = 0.744
3–6 months	9	13	
> 6 months	5	4	
Total number of TACE by lesion			
≤ 1	25	35	*P* = 0.456
> 1	4	2	
TACE procedure			
DEB-TACE	13	14	*P* = 0.566
Lipiodol-TACE	16	23	
Tumor number			
1	16	18	*P* = 0.219
2	8	6	
≥ 3	5	13	
Tumor diameter			
≤ 3 cm	15	17	*P* = 0.641
> 3 cm	14	20	
Tumor location			
right	18	22	*P* = 0.748
left	8	6	
both	5	9	
Vascular invasion			
Presence	8	6	*P* = 0.262
Absence	21	31	
Tumor differentiation			
Well/Mod	27	35	*P* = 1.000
Poor	2	2	

*Abbreviation*: *HCV* hepatitis C virus, *AFP* α-fetoprotein, *TACE* transarterial chemoembolisation, *LT* liver transplantation, *DEB* drug-eluting bead

Table 5 showed univariate and multivariate analysis of the pre-TACE radiological HCC appearance variables related to near-complete necrosis. Pre-TACE HCC with arterial phase hyper-enhancement was highly associated with near-complete necrosis upon histopathological analysis (*p* = 0.002). 35 of 54 patients (64.8%) with hyper-enhancement in arterial phase displayed near-complete necrosis, compared with 2 of 12 patients (16.7%) with hypo/iso-enhancement. Similarly, “typical enhancement” was highly associated with near-complete necrosis (*p* = 0.010). 27 of 37 near-complete necrosis lesions (73.0%) displayed this imaging feature. The rate of near-complete necrosis in HCC with smooth tumor margin was significantly higher than in HCC with lobulated or infiltrative margin [31/47 (66.0%) vs. 6/19 (31.6%) respectively; *p* = 0.011]. The radiological classification of HCC based on the enhancement pattern and tumor margin was also highly associated with near-complete necrosis. Type A lesions showed a significantly higher proportion of near-complete necrosis than type B/C lesions [35/51(68.6%) vs. 2/15(13.3%) respectively; *p* = 0.000]. The multivariate analysis showed that only the radiological classification of HCC was related to near-complete necrosis (RR 14.2, 95% confidence interval 2.9–70.6, *p* = 0.001).

**Table 5:** Univariate and multivariate analysis of the pre-TACE radiological appearances variables related to near-compete necrosis

Variables	Tumor necrosis < 90%	Tumor necrosis ≥90%	*P* value
Enhancement pattern in arterial phase			
hyperenhancement	19	35	0.002
Hypo/isoenhancement	10	2	
Heterogeneous enhancement			
Presence	17	23	0.770
Absence	12	14	
Typical enhancement			
Presence	12	27	0.010
Absence	17	10	
Intratumoral necrosis			
Presence	2	8	0.190
Absence	27	29	
Attenuation on precontrast phase			
Hypoattenuation	5	9	0.485
isoattenuation	24	28	
Tumor margin			
Smooth	16	31	0.011
Lobulated/infiltrative	13	6	
Tumor capsular			
Presence	9	13	0.726
Absence	20	24	
Radiological classification			
Type A	16	35	0.000^a^
Type B/Type C	13	2	

^a^Independently related to near-complete necrosis on the multivariate analysis (radiological classification RR = 14.2, 95% CI = 2.9–70.6, *p* = 0.001)

### Survival

**Fig. 2:**
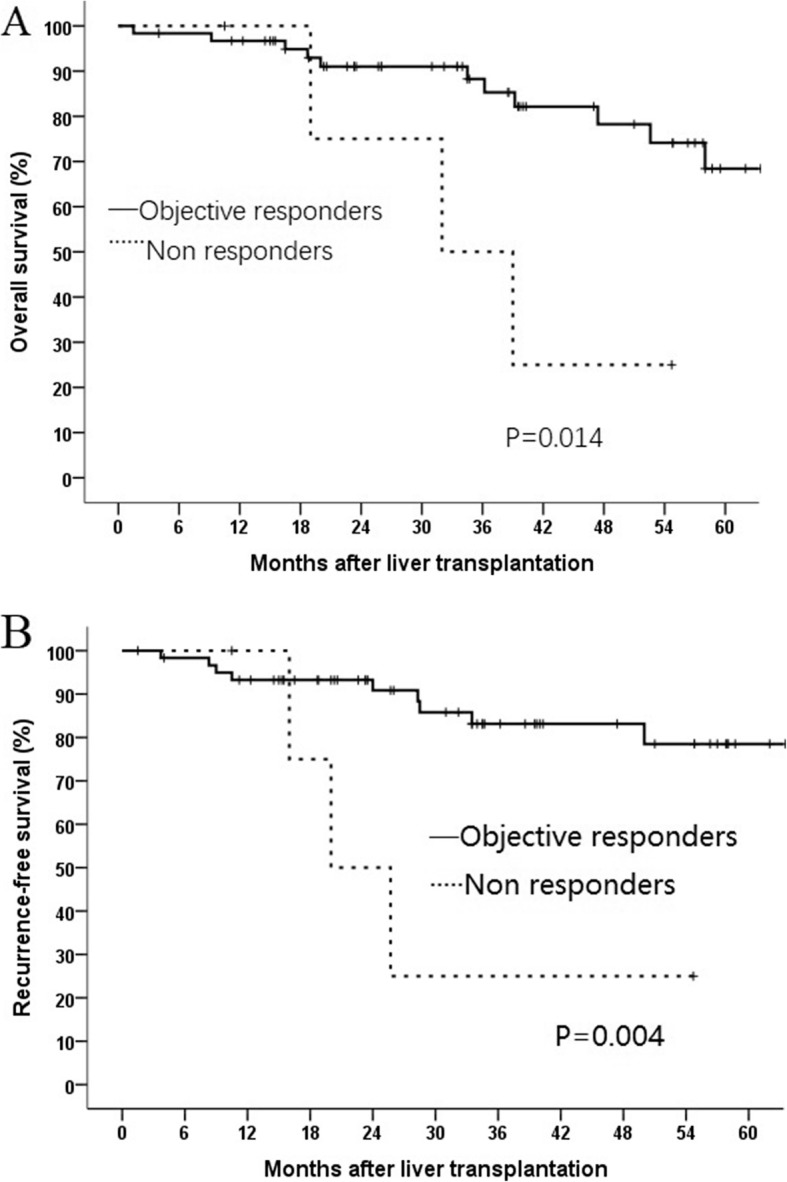
Kaplan–Meier curve comparing overall survival (**a**) and recurrence-free survival (**b**) between objective responders and non-responders according to the radiological response (mRECIST criteria) after transarterial chemoembolization (TACE)

For the 66 patients, the median post-LT follow-up was 72.8 months (range = 3–146 months). The 1-, 3-, and 5-year OS rates were 96.4, 85.1, and 66.5%, respectively. The 1-, 3-, and 5-year RFS rates were 93.6, 78.6, and 74.5%, respectively. Kaplan–Meier curves were constructed to measure the association between survival and TACE response. According to mRECIST, patients obtaining a CR or PR after TACE cycles are defined as objective responders (OR). Objective responders had significantly better OS and RFS than non-responders (*P* = 0.014 and *P* = 0.004, respectively). (Fig. 2).

## Discussion

Locoregional therapies (LRT) for HCC have emerged to treat unresectable disease or as a bridge to liver transplantation [5]. LRT include trans-arterial catheter therapies (TACE and Y-90 radioembolization), radiofrequency ablation and external beam radiation. TACE is considered the gold-standard LRT for unresectable, intermediate-stage HCC according to the BCLC classification [20]. While comparative studies revealed that TACE-based multi-modal treatments were safe and more beneficial than conservative management [21, 22], improved post-LT survival in patients undergoing pre-LT TACE remains equivocal [23]. Nevertheless, response to TACE has been established as a surrogate of tumor biology and instrumental to select patients for LT, since it has been observed that patients with a favorable response to treatment have optimal post-LT outcome, as opposed to those without a favorable response [24, 25].

Although the ultimate goal of LRT is to induce total tumor necrosis, the occurrence of this outcome is not achieved consistently and is subjected to a number of variables including tumor burden, tumor biology, number, type and technique of therapies. A number of studies have performed post-TACE histopathological analysis of explanted livers following LT in patients with HCC. Yao et al. reported complete tumor necrosis in 44% of patients [26], whereas Herber et al. reported complete tumor necrosis in only 16.7% of 42 patients [14]. In our study, the histopathological analysis of the explanted livers demonstrated a rate of complete necrosis of 31.8% (21 patients), comparable to previous reports on chemoembolization [27, 28]. Our number, however, might be toward the lower end of the reported range for studies based exclusively on selective TACE. Selective TACE has demonstrated higher rates of histopathological tumor necrosis compared to non-selective TACE. Golfieri et al. compared both lobar and selective TACE, obtaining complete tumor necrosis in 29.8 and 53.8% of cases, respectively (*p* = 0.013) [29]. Consistently, in a study assessing 109 lesions treated with selective TACE, Kwan et al. have shown 62 cases (57%) of complete histopathological tumor necrosis with an overall mean tumor necrosis of 70% [15].

Previous reports have shown that the rate of complete necrosis may vary widely, according to the heterogeneity of the patient populations, the diameter of the lesions, the number of procedures performed in each patient, and the time between the first TACE and the actual OLT. Compared to the sample studied by Kwan et al., our population experienced a shorter time from the first TACE to OLT (with a mean of 197 and 108 days, respectively). Highly necrotic lesions are less prone to either local or systemic recurrence and therefore allow patients to remain on the waiting list for longer periods of time. Furthermore, the mean lesion per patient in our population was 2.09, compared to 1.3 in their study. Nevertheless, even though these variables were regarded as statistically significant in recent studies [8, 15], none of them have been shown to influence the extension of necrosis in ours.

In order to further determine what factors can impact HCC response to TACE, we classified HCC lesions into three categories based on radiological appearance. Notably, the odds of near-complete necrosis for a lesion demonstrating avid enhancement on arterial phase (69%) was approximately 2 times higher than that of a lesion showing only mild to moderate enhancement (37%) (*p* = 0.002). For further analysis of the data, we evaluated each type of hyper enhancement individually, finding that only typical enhancement (*p* = 0.010), but not heterogeneous enhancement (*p* = 0.770) was associated with favorable TACE outcome. Such contrast might be explained by the dissimilar degree and type of vascular flow found in radiologically different tumors. Similar differences were found regarding tumor margins: 67% (31 patients) of the tumors with smooth margins showed near complete necrosis, compared to only 31% (6 patients) of the tumors with lobulated/infiltrative tumor margins (*p* = 0.011). Our proposed tumor classification, which includes both parameters, displayed even larger variances between groups. While only 13% (2 patients) of type B and C tumors showed near complete necrosis in the explant analysis, the proportion was 5 to 6 times higher (69%, 35 patients) in their type A counterparts (*p* = 0.000). These findings suggest that the use of parameters related to enhancement and margin may aid in the prediction of the TACE outcome, and therefore, it may be helpful to consider both variables concomitantly before subjecting patients to a procedure.

A lower Child-Pugh score was the only non–imaging-related variable assessed in this study that showed statistical significance regarding TACE response (*p* = 0.045). This association is consistently found in the studies [30, 31], and may be related to the more aggressive treatment regimen that patients with a better functional liver status were able to tolerate, compared to that which was used in the context of more advanced liver disease. As expected, no other demographic considered in the study, either in clinical or laboratory data, including Milan criteria (*P* = 0.149), tumor location (*P* = 0.798) and AFP levels (*P* = 0.444), were significantly associated with near-complete necrosis at the *P* < 0.05 level.

Finally, taking advantage of the fact that LT offers the possibility of assessing histological tumor necrosis after treatment with TACE, we have been able to compare mRECIST outcomes with histopathological alterations. If specific radiological features accurately matched the histologic status of the HCC, CT after TACE would become a useful tool to select or exclude patients from transplantation. We observed a satisfactory correlation between CT and pathologic data regarding the degree of tumor necrosis (63%), with an underestimation of tumor response in 18 (27%) patients and an overestimation in 6 patients (9.1%). Based on over 138 nodules in 66 patients, our results suggest that CT may tend to underestimate tumor necrosis, with a particularly low positive predictive value (55.2%), but may offer an acceptable level of accurancy in detecting complete necrosis (76.2%). Similar findings were published by Bargellini et al.*,* who observed a coorelation between mRECIST and pathology in 120 of 178 patients (67.4%), with 19 cases (10.7%) of underestimation and 39 cases (21.9%) of overestimation of tumor response to CT [8]. Since only a minority of patients qualify for resection or liver transplantation, experience correlating imaging features of HCC lesions after TACE with findings from pathological analysis has been restricted [14, 32, 33].

Limitations noted in this study included: use of varying CT equipment and TACE regimens, and treatment of patients which took place over a broad timeframe. Furthermore, due to the artifacts generated by the dense accumulation of ethiodized oil, the estimation of tumor necrosis and recognition of remaining viable enhancing tumor can be misleading, especially after Lipiodol-TACE [34]. Finally, this cohort only included a restricted number of patients who underwent both TACE and OLT. The small number of cases forced us to put together type B and type C tumors in one group before carrying out the analysis, preventing us from obtaining results for each type separately. It is also possible that the restriction in patients was partly responsible for the relatively low rate of a CR reported and the low incidence of repeated TACE, compared to the overall population of patients with HCC, especially those who have undergone selective TACE.

## Conclusions

In conclusion, our study findings indicate that well circumscribed HCC lesions with arterial phase hyper enhancement are more susceptible to TACE than lesions with arterial phase iso- or hypo-enhancement and lesions with infiltrative appearance. By using this interpretation, CT could allow for the narrowing of the selection criteria for TACE, as well as for increasing overall desirable outcomes and prognosis of LT following bridging procedures. It is highly desirable to select patients with biologically favorable tumors for LT in order to avoid the use of scarce organs for questionable outcomes. Nevertheless, more and larger studies are required to validate the role and relevance of these imaging parameters.

## Data Availability

The datasets used and/or analysed during the current study are available from the corresponding author on reasonable request.

## Abbreviations

BCLC: Barcelona Clinic Liver Cancer
CR: Complete tumor response
HBV: Hepatitis B virus
HCC: Hepatocellular carcinoma
HCV: Hepatitis C virus
LRT: Locoregional therapy
LT: Liver transplantation
MDCT: Multidetector computed tomography
mRESIST: Modified response evaluation criteria in solid tumors
NPV: Negative predictive values
PD: Progressive disease
PPV: Positive predictive values
PR: Partial response
SD: Stable disease
TACE: Trans-arterial chemoembolization
